# P-2090. Using MRSA Nares PCR to Predict MRSA Purulent Skin and Soft Tissue Infections

**DOI:** 10.1093/ofid/ofae631.2246

**Published:** 2025-01-29

**Authors:** William Greene, Sadaf Aslam

**Affiliations:** University of South Florida, Keystone, South Dakota; University of South Florida Morsani College of Medicine, Tampa, Florida

## Abstract

**Background:**

Skin and soft tissue infections (SSTI) are divided into purulent vs non-purulent with the former necessitating the need for methicillin resistant *staph aureus* MRSA antimicrobial coverage per IDSA guidelines for management of SSTI. MRSA nares PCR screening for MRSA pneumonia has demonstrated high negative predictive value (NPV) (98.1%) for MRSA associated community-acquired pneumonia (CAP) and hospital-acquired pneumonia (HAP). Studies evaluating MRSA nares PCR to predict MRSA infection involving other anatomical sites including skin/soft tissue have shown mixed results. This retrospective cohort study seeks to further evaluate the utility of MRSA nares PCR in predicting MRSA associated purulent SSTI including in relation to anatomical site of the skin infection (above the waist line vs below the waist line).

**Figure:**
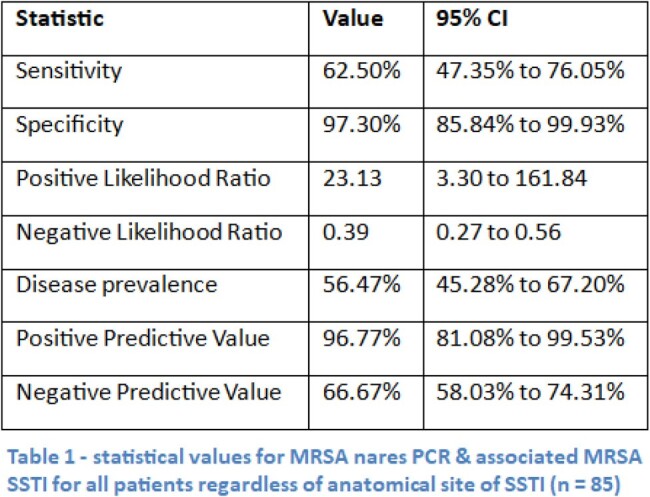

**Methods:**

We performed a retrospective medical record review of patients admitted to the hospital or evaluated in the emergency department with SSTI between 1/1/2015-11/30/2022. Patients 18 years or older with a diagnosis of purulent cellulitis were included if they had a MRSA nares PCR result and a positive culture for *staph aureus* from either a purulent wound culture swab or an abscess incision/drainage culture. Medical records were reviewed to confirm a diagnosis of purulent cellulitis including physician documentation and pictures of associated skin infections when available. The anatomic site of the SSTI were recorded.

**Figure:**
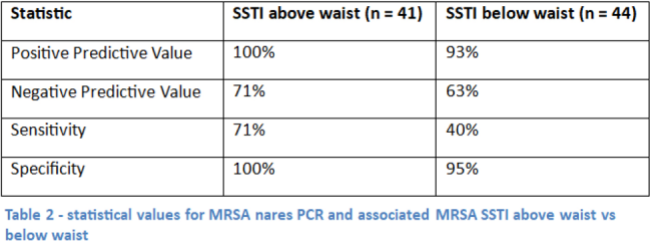

**Results:**

85 patients were included in the final analysis. MRSA nares PCR for MRSA SSTI had a negative predictive value (NPV) of 66.7% and positive predictive value (PPV) of 96.8%. The sensitivity and specificity of MRSA nares PCR for MRSA SSTI was 62.5% and 97.3% respectively. The NPV of MRSA nares PCR for MRSA SSTI above the waist was 71% vs 63% below the waist.

**Conclusion:**

Our results are consistent with other studies showing high specificity and low sensitivity for MRSA nares PCR in predicting MRSA SSTI. However, the NPV was much lower in this cohort compared to other studies. These results do not support the use of MRSA nares PCR to rule out MRSA SSTI. Clinicians should continue to use clinical exam (purulent vs non-purulent) and microbiologic data when available to guide antimicrobial coverage of MRSA when treating patients with SSTI.

**Disclosures:**

All Authors: No reported disclosures

